# Supplementary material for: Mid-trimester cervical length not associated with HIV status among pregnant women in Botswana
Source: PLoS One. 2020 Mar 11;15(3):e0229500. doi: 10.1371/journal.pone.0229500 (PMC7065819; doi:10.1371/journal.pone.0229500)
Supplement: Attachment — Submitted filename: CL_PLOS1_ResponsetoReviewers_1.4.20.docx [file pone.0229500.s002.docx]

January 4^th^, 2020

Dear *PLOS ONE* Reviewers,

Thank you for your thoughtful review of our manuscript “Mid-trimester cervical length not associated with HIV status among pregnant women in Botswana”. We appreciate your continued consideration of our manuscript for publication in *PLOS ONE* and believe the manuscript has been strengthened by incorporating this feedback*.* Please see the revised manuscript attached, and the numbered responses to your comments below.

1. *Are the outcomes of the pregnancy (maternal) available? If yes, provide information about the gestational age of delivery and the numbers delivering <37 weeks.*

*Reviewer #1 full comment: “This manuscript represents an excellent study attempting to determine the rate of midtrimester cervical shortening among women with HIV in Botswana. The authors propose to use this evaluation as a proxy measure for understanding the underlying mechanism for an increased preterm birth rate among women with HIV on ART. The scientific design was reasonably rigorous for the question asked, and the manuscript is well prepared.

My chief issue with this manuscript is that there is no data reported regarding whether any of the subjects in the trial actually went on to have a preterm birth. This data may be easily obtainable since this is a nested study within a large longitudinal cohort. Alternatively, because women with short cervix were referred elsewhere, this data is unavailable. It is possible that the authors do not present this information because the purpose of the study was to determine the rate of cervical shortening, rather than the correlation between cervical shortening and preterm birth in this population. However, given that they are speculating at a cause of preterm birth among women with HIV, their relatively small sample population may simply have missed all the women who actually went on to preterm birth. In this case, they are unable to conclude that cervical shortening does not contribute to preterm birth.

The manuscript would be strengthened by reporting gestational age at birth for the cohort. If this population did indeed have an earlier GA at birth among women with HIV, then the authors conclusions are substantiated. If this data is unavailable, the discussion should include a more robust description of the limitations of this population sample in determining contributors to preterm birth. The sentence currently included in the discussion, "Our study was not powered to show a difference in PTB by exposure categories in the cohort, including differences between HIV-infected and HIV-uninfected women." is insufficient in demonstrating to the reader these nuances.”*

Response 1: Outcomes data was available for only 68% of our cohort, and we included the details in the revised manuscript as suggested. In this subset of women, eight out of 122 women had a preterm birth (6.6% overall; 2/30 HIV-infected women, 6.7%; 6/92 HIV-uninfected women, 6.5%; p=0.59), and one of these 8 women had a short cervix <25mm. These are interesting results, but our ability to draw significant conclusions from this data is limited.

Our group has previously reported on the increased risk of preterm birth among pregnant women infected with HIV in our population in Botswana.^1–4^ The current study was designed to evaluate the cervical length distributions between HIV-infected and HIV-uninfected women rather than demonstrate a link between cervical shortening and preterm birth as has been previously established.^5^ We did not observe a difference in the cervical length distributions by HIV status. While cervical shortening heightens the risk for preterm birth, not all preterm births are preceded by cervical shortening, as was the case for our participants. Among the eight women with preterm births in our cohort, only one had a mid-trimester cervical length less than 25mm. Through additional post hoc power analysis, we determined that an increase in our sample size by 50% in each group would yield similar results. In conclusion, our study suggests that the increased risk of PTB observed among HIV-infected women in Botswana is unlikely to be associated with mid-trimester cervical shortening. Further research is needed to understand the mechanism of PTB in HIV-infected women receiving ART.

Response 1 References:

1.	Zash, R. *et al.* Comparative Safety of Antiretroviral Treatment Regimens in Pregnancy. *JAMA Pediatr.* **171**, e172222 (2017).

2.	Shapiro, R. L. *et al.* Antiretroviral regimens in pregnancy and breast-feeding in Botswana. *N. Engl. J. Med.* **362**, 2282–2294 (2010).

3.	Powis, K. M. *et al.* Increased risk of preterm delivery among HIV-infected women randomized to protease versus nucleoside reverse transcriptase inhibitor-based HAART during pregnancy. *J. Infect. Dis.* **204**, 506–514 (2011).

4.	Chen, J. Y. *et al.* Highly active antiretroviral therapy and adverse birth outcomes among HIV-infected women in Botswana. *J. Infect. Dis.* **206**, 1695–1705 (2012).

5.	Iams, J. D. *et al.* The length of the cervix and the risk of spontaneous premature delivery. National Institute of Child Health and Human Development Maternal Fetal Medicine Unit Network. *N. Engl. J. Med.* **334**, 567–572 (1996).

2. *“Table 2 and 2a: The length <30 mm is not needed since >25 mm is considered normal.”*

Response 2: We agree, and removed this line in the table.

3. *“The information about ART can be in the text of the manuscript and not needed in Table.”*

Response 3: This data is available in the text, and was removed from the table as suggested.

4. *“Please amend your current ethics statement to address the following concerns:
   a) Did participants provide their written or verbal informed consent to participate in this study?
   b) If consent was verbal, please explain i) why written consent was not obtained, ii) how you documented participant consent, and iii) whether the ethics committees/IRB approved this consent procedure.”*

Response 4: All volunteers for this study signed an approved written consent in Setswana or English (per language preference) prior to participation in the study. We clarified this in the manuscript on page 9 under “Ethics Approvals”.

5. *“We note that you have indicated that data from this study are available upon request. PLOS only allows data to be available upon request if there are legal or ethical restrictions on sharing data publicly. For information on unacceptable data access restrictions, please see http://journals.plos.org/plosone/s/data-availability#loc-unacceptable-data-access-restrictions.”*

Response 5: The de-identified dataset for this study, including the outcomes data, was uploaded to the PLOS ONE site as a supporting file. Please see the attached file.

6. *“Commentary regarding mechanism of progesterone use in HIV infected women with normal cervical length appears out of place in this manuscript as this is an ultrasound study, not a therapeutic study. If the subjects in the trial did get vaginal progesterone and the authors think this confounded the preterm birth rate in the cohort, that should be explicitly said. Otherwise I would remove this section.”*

Response 6: To limit any confusion, we removed the paragraph in the discussion about vaginal progesterone. At this time, vaginal progesterone is not offered in Botswana as part of a prevention strategy to decrease risk of preterm birth, regardless of HIV status.

7. *“The acceptability data is interesting, and should be highlighted in the abstract if there is word count to support it.”*

Response 7: Thank you for acknowledging the importance of the transvaginal cervical length acceptability data. We highlighted the acceptability data in the abstract as you suggested.

In summary, thank you for your thoughtful analysis of our paper. We hope you will continue to consider our manuscript for publication in *PLOS ONE*.

Please do not hesitate to contact me if you have any further questions at [iliff@tuftsmedicalcenter.org](mailto:iliff@tuftsmedicalcenter.org) or at 617-835-4483.

Sincerely yours,

Ingrid Liff, MD

Assistant Professor, Tufts University School of Medicine

Division of Maternal Fetal Medicine, Department of Obstetrics and Gynecology

Tufts Medical Center

800 Washington Street

Boston, Massachusetts

02111
